# P-388. On-time injection adherence and persistence among people with HIV on long-acting cabotegravir/rilpivirine

**DOI:** 10.1093/ofid/ofaf695.605

**Published:** 2026-01-11

**Authors:** Sharan Yadav, Yotam Arens, Elizabeth Lee

**Affiliations:** Weill Cornell Medicine, New York, New York; Weill Cornell Medicine, New York, New York; New York Presbyterian, New York, New York

## Abstract

**Background:**

Long-acting cabotegravir-rilpivirine (LA-CAB/RPV) injections may help people with HIV (PWH) overcome adherence barriers to daily oral antiretroviral therapy. Challenges to implementation of LA-CAB/RPV programs include intensive pharmacy and nursing support and frequent patient visits. Real-world data on adherence to on-time injections and persistence on therapy is limited.

**Figure:**
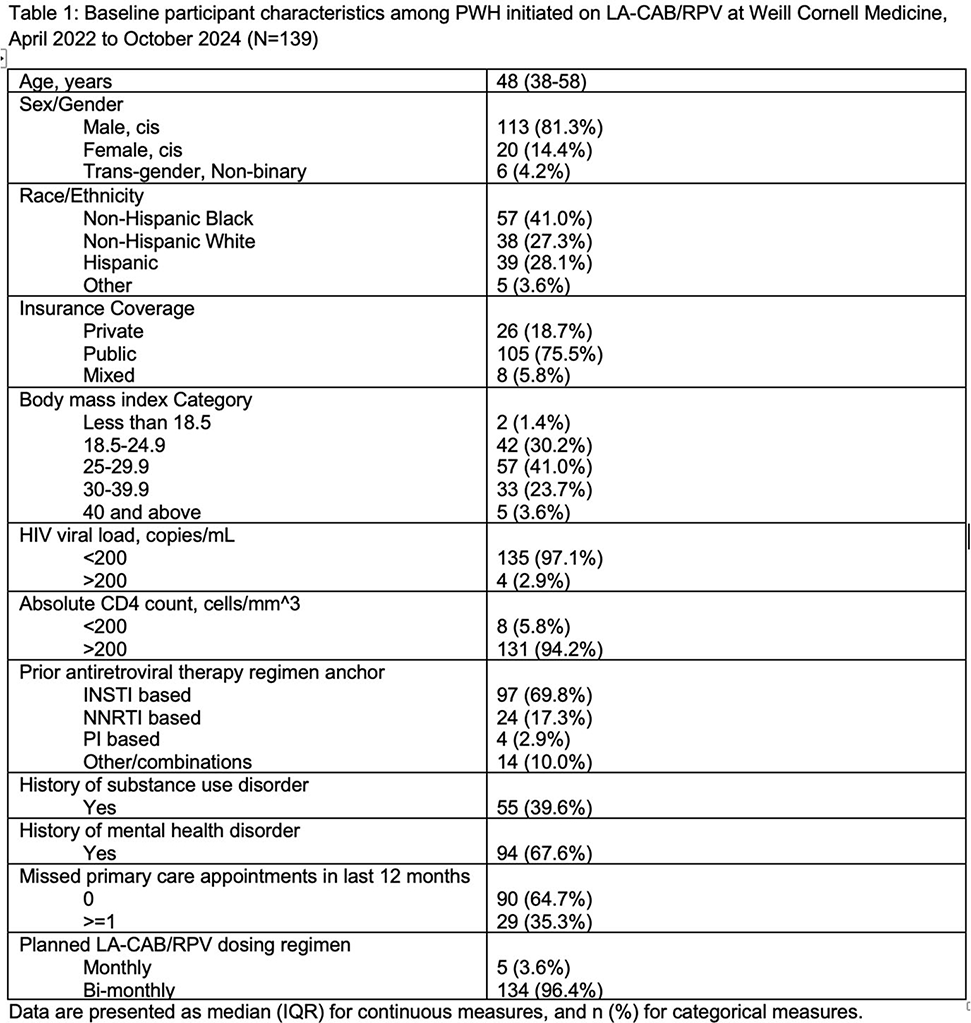

**Figure:**
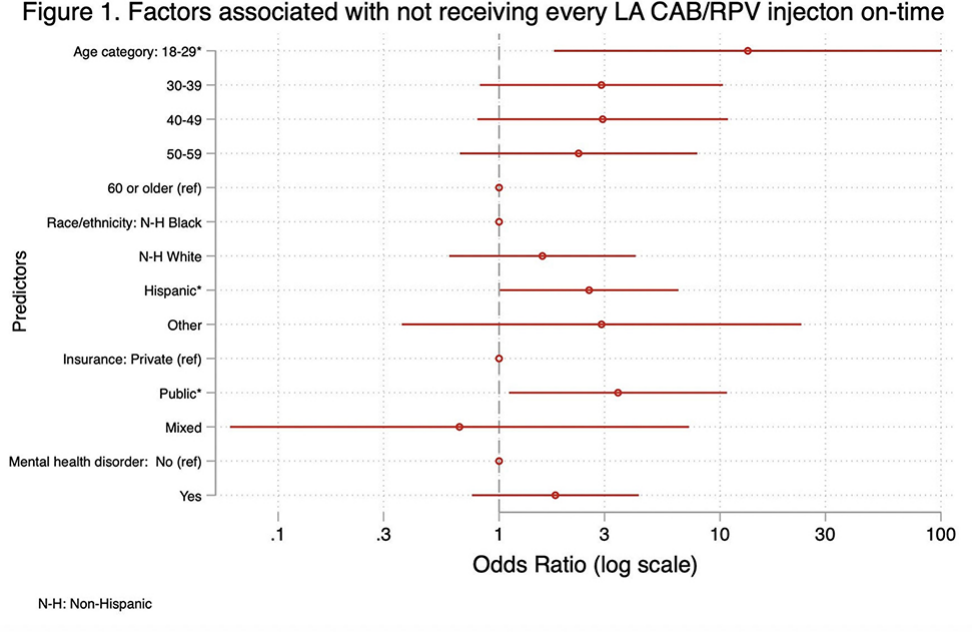

**Methods:**

We performed a retrospective cohort study using chart review of adult PWH at Weill Cornell Medicine who received ≥ 1 LA-CAB/RPV loading injection as part of routine care between APR2022 and OCT2024. Follow up time began at first loading injection; censoring occurred at LA CAB/RPV discontinuation, last visit, or end of analysis period, ARP2025. On-time injections were defined as follow up injections +/- 7 days of target injection date (28 days from last injection for second loading or Q4W dosing, or 56 days from last injection for Q8W dosing). We calculated the proportion of all injections administered on-time,the proportion of patients who received all injections on-time, and rates of LA-CAB/RPV discontinuation. Multivariable logistic regression was used to estimate predictors of missed/delayed injections. We measured rates of viral suppression (VL < 50 copies/mL) at LA-CAB/RPV discontinuation or last follow up.

**Results:**

A total of 139 patients met study criteria. Patients were predominantly male (81%) and non-Hispanic Black (41%), median age was 48 (IQR 38-58), and 94% had baseline VL < 50 copies/mL. Patients received a median of 10 (IQR 5-15) injections, and 28 (20%) discontinued LA CAB/RPV during follow up. Of 1426 planned injections, 1398 (98%) were administered and 1357 (95%) were administered on-time. Eighty eight (63%) patients had every injection administered on-time. Factors associated with not receiving every injection on time were younger age, 18-29 (OR 13.3, p=0.01), public insurance (OR 3.5, p=0.03), and Hispanic ethnicity (OR 2.6, p= 0.05). A total of 129 (93%) patients had VL < 50 copies/mL at last measurement.

**Conclusion:**

In this real-world cohort, 95% of LA CAB/RPV injections were administered on time. While only 63% of patients had every injection administered on-time, maintenance of viral suppression and persistence on LA-CAB/RPV therapy for > 12 months were high.

**Disclosures:**

All Authors: No reported disclosures

